# Remote Assessment: Origins, Benefits, and Concerns

**DOI:** 10.3390/jintelligence11060114

**Published:** 2023-06-09

**Authors:** Christy A. Mulligan, Justin L. Ayoub

**Affiliations:** 1 Derner School of Psychology, Adelphi University, 1 South Avenue, Garden City, NY 11530, USA; 2 Nassau BOCES, 71 Clinton Road P.O. Box 9195, Garden City, NY 11530, USA

**Keywords:** remote assessment, teleassessment, virtual assessment

## Abstract

Although guidelines surrounding COVID-19 have relaxed and school-aged students are no longer required to wear masks and social distance in schools, we have become, as a nation and as a society, more comfortable working from home, learning online, and using technology as a platform to communicate ubiquitously across ecological environments. In the school psychology community, we have also become more familiar with assessing students virtually, but at what cost? While there is research suggesting score equivalency between virtual and in-person assessment, score equivalency alone is not sufficient to validate a measure or an adaptation thereof. Furthermore, the majority of psychological measures on the market are normed for in-person administration. In this paper, we will not only review the pitfalls of reliability and validity but will also unpack the ethics of remote assessment as an equitable practice.

## 1. Introduction

In March 2020, the doors closed indefinitely for many schools across the nation due to the COVID-19 pandemic. In reflection, the world seemed uncertain, as hospitals and first responders were tested to their limits, and many children’s education was paused. The hiatus in education has further highlighted the educational inequities that existed pre-COVID-19 and essentially widened the educational gap. This also posed many legal questions for many districts regarding how to provide services to students in special education, as well as how to complete outstanding psychoeducational evaluations within the designated timelines. The Office of Civil Rights (OCR) provided some guidance and extensions for initial and re-evaluations. The OCR recommended that parents and the district mutually agree upon the length of the extension, although this was not clearly defined (OCR of Special Education and Rehabilitative Services 2020). Many districts aiming for compliance turned to remote assessment to comply with the Individuals with Disabilities Education Improvement Act (IDEIA 2004). 

Telehealth services have increased over the past decade (Love et al. 2019) and will likely increase in the future (Darling-Hammond et al. 2020; Goddard et al. 2021). However, remote assessment is not appropriate or accessible for all children, or for all referral questions of suspected disability. Although remote assessment seems to be a viable solution to address the needs of children, literature on the reliability and validity is still growing and practitioners should therefore use these techniques with caution. 

The role of the school psychologist is multifaceted, but one area that never wavers is the necessity to follow the IDEIA and corresponding laws of their state. School psychologists were tasked with meeting timelines for evaluations and abiding by the Child Find mandate, which is a part of special education law emphasizing that schools are required to locate, identify, and evaluate all children with disabilities from birth through age 21. (20 U.S.C. 1412(a)(3)). The provision of mandated psychological services was paramount for school psychologists to address, yet it was nearly unachievable to maintain compliance with federal and state regulations considering the mass school closures. Furthermore, the OCR was clear that if schools were offering educational opportunities for children in general education, they must continue to provide them for students with disabilities (USDOE 2020), therefore compelling districts to provide services and evaluate children suspected to have a disability through teleassessment. 

School psychologists were in unchartered territory during the COVID-19 pandemic, as they had never been placed in the position to evaluate and provide continuity of services remotely. This paper will highlight some of the approaches schools used to meet the needs of their students, the strengths and weaknesses of remote assessment as well as the obstacles to providing equitable and ethical practice in teleassessment. Social justice and ethical concerns will be emphasized so that school psychologists are cognizant of the advocacy necessary to address the needs of the marginalized groups of children who were most affected by the pandemic. 

## 2. Guidance from Professional Organizations on Remote Assessment

Prior to the COVID-19 pandemic, there was little need for remote assessment. Telehealth was in its infancy, and school psychologists did not have assistance or graduate training to direct them. However, as the COVID-19 pandemic paused essential services this catalyzed an interest in teleassessment, as testing companies offered resources and recommendations to adapt tests to this modality (Pearson 2021), professional organizations released guidance (see Table 1) on how to effectively and ethically conduct (or not conduct) teleassessments (California Association of School Psychologists 2020; APA 2020), and independent researchers began exploring the reliability and validity of measures administered remotely (Hamner et al. 2022; Wright 2020). The APA Div 12 (Society of Clinical Psychology) developed guidelines for psychologists conducting remote psychological assessments. The principles are meant to be considered as a whole, with no one principle allowing psychologists to modify test administration (APA 2020). The goal of the principles is to guide the practice of psychologists when face-to-face assessment is limited. If administration procedures need to be altered, psychologists must also consider how these alterations may impact the test data, e.g., do the results yield an accurate representation of the individual’s abilities despite modified administration? Lastly, psychologists should practice this adjusted administration prior to seeing their examinee (APA 2020). 

Following these guidelines and considering the recommendations made by APA, NASP, IOPC, and test publishers will help to ensure an ethical, sensible, and thoughtful remote evaluation. As technologies in remote assessment and test publication advance, examiners will have the option of choosing which modality to administer (Kaufman and Kaufman 2022a). Currently, there are several assessments on the market exclusively intended to be administered remotely and this number is expected to grow.

## 3. Remote Assessment: Strengths and Challenges

Traditionally, psychological assessment is administered face-to-face between the examiner and the examinee, in a quiet location, free of distractions. In fact, some parts of the assessment process would be very challenging to administer remotely, e.g., Block Design on the Wechsler Intelligence Scale for Children- Fifth Edition (WISC-V; Wechsler 2014), as the examiner typically places the blocks in front of the child in a standardized format. However, in a small field study conducted in 2019, researchers studied the agreement of scoring between face-to-face administration and remote administration on the WISC-V. They found very high correlations for the WISC-V index scores, ranging from .981 to .997, with the full-scale IQ correlated at .991 (Hodge et al. 2019). Although the sample was small, this suggests scores may not be influenced by the administration format. Furthermore, there have been larger studies conducted that have demonstrated similar evidence of no significant impact of teleassessment versus face-to-face administration. The assessments included in this study were cognitive assessments and included the Woodcock–Johnson IV Test of Cognitive Ability (WJ-IV-Cog; Schrank et al. 2014a), the Reynolds Intellectual Assessment Scales, Second Edition (RIAS-2; Reynolds and Kamphaus 2015), and the WISC-V (Wright 2018a, 2018b, 2020). These studies offer preliminary evidence of score equivalency between administration formats; however, more research is necessary to fully validate adaptations of current cognitive assessments that were intended for in-person administration. Moreover, what could be lost or hindered by remote assessment are the rich behavioral observations of how the child approached the task or nuanced levels of frustration, which may be absent if administered virtually.

Hitherto, studies on teleassessment have typically focused on neuropsychological measures in adults. This research base lends support to the use of neuropsychological measures via teleassessment in adults, indicating score equivalency (Brearly et al. 2017; Galusha-Glasscock et al. 2016; Temple et al. 2010), diagnostic agreement (Loh et al. 2007) and diagnostic accuracy (Wadsworth et al. 2016). Brearly et al. (2017) conducted a meta-analysis comparing in-person versus remote administration of adult neurocognitive tests and found consistency of scores across administration methods. Nonetheless, it is important to acknowledge that just two of the twelve studies included in the meta-analysis had participants with a mean age below 65. Studies in which the participant’s average age exceeded 75 indicated heterogeneity of scores between administration methods. 

While much emphasis has been placed on how to adapt traditional face-to-face assessment remotely, there are assessments that were developed to be administered using an online format. One such assessment is the MEZURE instrument, which is a cognitive measure of ability for ages 6 through adulthood; it is fully administered and scored online, which will invariably reduce administration and scoring errors (Assessment Technologies Inc. 2021); in fact, the examiner has only a minimal role in administering the MEZURE (Dombrowski et al. 2022). This assessment provides measures of crystallized and fluid intelligence as well as processing speed, memory with distractions, social perception, and a measure of stress tolerance for the adult population. According to the clinical manual, the MEZURE aligns with Cattel–Horn’s Gf-Gc theory of cognitive abilities (Cattell and Kuhlen 1963; Horn 1965). The psychometric properties are included in the clinical manual (Assessment Technologies Inc. 2021) and include reliability as well as an exploratory factor analysis of validity. However, a limitation is that the criterion-related validity is a correlation between the overall score of the MEZURE and the WISC-III, which is quite outdated, and the test is plagued with other validity concerns. It seems at minimum that the MEZURE should be updated to ensure correlational data are current to the latest edition of the Wechsler cognitive assessment to avoid the Flynn effect (Flynn 1984). Lastly, the WISC-III is also an instrument exclusively used for children, aged 6–16; yet, MEZURE claims their assessment operates well for adults. Due to internal validity issues, MEZURE should be used and interpreted with caution. 

CogniFit general cognitive assessment (CAB) is a measure of general cognitive well-being in children aged 7–adulthood. The website describes this assessment as a neurocognitive test that is used to understand an examinee’s general cognitive state. The Cognifit is fully administered online and is a computerized cognitive assessment. Its intended use is for any private or professional user to be able to easily access this cognitive assessment. This online cognitive test shows how people score in concentration/attention, memory, reasoning, planning, and coordination (Cognifit n.d.). Although Cognifit’s subtests seem to measure what they are supposed to measure, this assessment is not without weaknesses, one being the difficulty in interpreting the computer-generated report, as the assessment has an unusual scoring system. The second problem is the psychometrics, as the clinical manual only lists the reliability, not the validity (Cognifit n.d.). Due to these psychometric pitfalls, this assessment should not be used by professional or school psychologists. 

## 4. Reviewing Records, Interviewing Key Informants, Observing Students and Administering Tests (R.I.O.T.)

R.I.O.T. is an important consideration for remote assessment, and a way to conceptualize the functioning of a child using a variety of data points (Leung 1993). Many school districts relied on this method of assessment, oftentimes in the absence of administering tests. Leung (1993) argued that school psychologists should be cautious of “doubling down” on data collected, meaning that these clinicians should not overload data from one method of collection (p. 1). To combat this issue, cross-validating the findings with data gathered using other methods is endorsed (Leung 1993). If we use this approach, we might complete a classroom observation on a child, compare this to what the teacher observes in the classroom, and interview the parent to understand how the child functions at home. This can be strengthened by considering data from rating scales and, finally, the assessment, or “testing.”

The World Health Organization officially declared an end to the COVID-19 global health emergency and the United States allowed its COVID-19 public health emergency state to end on 11 May 2023 (Gumbrecht et al. 2023). Therefore, there is less of a need for remote assessment, and it has been de-prioritized in relation to other pressing concerns in the schools. However, because many schools used aspects of R.I.O.T. to address their assessment needs, we wanted to highlight the strengths and weaknesses of this approach. 

There are several strengths of the R.I.O.T., with one being that it endorses gathering multiple sources of data to complete a comprehensive psychological evaluation. This is in line with meeting the legal mandate to use a variety of evaluation tools and approaches and not rely on any single source of data when making high-stakes educational decisions about students (IDEIA 2004). A second strength of this method of evaluation is it engages the child study team (CST) to collaborate together to understand the needs of the child and advocate for them. Lastly, it compels the school psychologist to go through the process of interviewing multiple informants, perhaps gleaning a perspective that they would not originally have had. During this interview process, the interviewer may have an easier time accessing potential interviewees, as this may be conducted remotely. Parents/guardians would have the opportunity to schedule a meeting during a lunch break, allowing for greater flexibility during their day without the obligation to travel. 

One potential struggle with using R.I.O.T. for remote assessment is conducting the observation. It may be difficult to observe a child in their natural environment remotely. Moving out of the view of the camera and leaving the room are both scenarios that make an observation via remote assessment undesirable. When observing a child, one wants both a reliable and valid observation, which would be difficult to accomplish considering the freedom to move around is limited, and the child would most certainly know they were being observed, which could impact their behavior (Adair 1984). However, there are methods of achieving a more organic observation, such as engaging a parent to video-record their child and provide the footage to the clinician (Nazneen et al. 2015). 

Although we believe the R.I.O.T. is a strong approach to conceptualizing and evaluating students, some researchers have endorsed taking the “T” out of R.I.O.T. (Hass and Leung 2021). Although schools and districts can arguably review records, and interview stakeholders, the last two processes in the R.I.O.T. acronym are more complex to conduct remotely. If a district only relies on R.I.O., and ignores the testing piece, we argue this can be problematic. According to NASP Guiding Principle II.3 Responsible Assessment and Intervention Practices, it is permissible for school psychologists to make recommendations based on a review of records; however, they need to use a representative sample of records, and explain the “basis for, and limitations of their recommendations” (NASP 2020b, p. 47). Unfortunately, all too often a comprehensive review of records is insufficient due to the dearth and age of the information being reviewed to make any definitive educational conclusions. This may be especially common in states where the student-to-school psychologist ratio is well over the recommended 1:500 ratio (NASP 2021). For example, if a child had an initial evaluation in 3rd grade, a reevaluation with no new testing in 6th grade, and then a R.I.O. evaluation in 9th grade, a team could potentially be making a high-stakes decision about a student based on six-year-old assessment data, which is incomplete at best and irresponsible at worst.

School psychologists know that there are many variables that can impact cognitive abilities over time, e.g., socioeconomic status, and poor educational background (Carneiro and Heckman 2003), and a review of records is inadequate to determine continued eligibility, especially if records are old and testing has not been updated. Therefore, we argue it is best practice to re-evaluate with new testing as this is a significant piece of special education identification. 

## 5. Reliability and Validity of Remote Assessment with Children

Research on teleassessment in children has largely taken the form of equivalence studies. In an unpublished white paper, Wright (2018a) used a case–control match design to investigate the score equivalence between in-person and remote administration of the Reynolds Intelligence Assessment Scales-Second Edition (RIAS-2; Reynolds and Kamphaus 2015) with a sample of 104 children. The results of the study revealed that, for the four core RIAS-2 subtests, mean score differences were not statistically different across administration modes. Additionally, effect sizes were small. However, participants assessed in person scored significantly higher than participants assessed remotely on speeded tasks. This effect was only observed in participants aged 7 and younger, with the author positing that this could be due to the fact that voluntary attention improves developmentally with age (Wright 2018a). Based on the unpublished white paper, the RIAS-2 Remote has been released (Reynolds et al. 2020). There is limited information provided by the publisher other than an equivalency study, and there were no new norms developed. Although some would suggest that equivalency studies render scores interchangeable between in-person and remote assessment, we argue that there are newly released as well as forthcoming instruments that have gone undergone more rigorous validation procedures that may be a better choice when conducting remote assessment.

In a separate case–control match design with a sample of 240 children comparing scores between in-person and remote administration of the Woodcock–Johnson IV (WJ IV) cognitive (Schrank et al. 2014a) and achievement (Schrank et al. 2014b) tests, the results indicated no significant differences and minimal effect sizes between administration modes in cluster and individual test scores (Wright 2018b). Using a similar design, Wright (2020) examined score equivalence between administration modes in a sample of 256 children using the WISC-V (Wechsler 2014) and found no differences (using confidence interval bounds) in index or subtest scores between in-person and remote administration formats. Nonetheless, it was observed that participants in the traditional in-person format scored significantly higher than participants in the remote format on the letter–number sequencing subtest (Wright 2020). 

While these studies contribute significantly to our understanding of teleassessment, a few limitations should be considered. Firstly, these studies included nonclinical samples, and it must be determined how clinically referred children will respond to remote testing. Secondly, the remote condition in these studies was conducted on-site with a proctor. The amount of control exercised during the study eliminates possible sources of construct irrelevant variance (Farmer et al. 2020a). This limits the generalizability of the findings, as this level of control may not be feasible when examinees are assessed in more organic environments, e.g., their homes. 

Hamner and colleagues (Hamner et al. 2022) sought to address these research questions. They conducted a retrospective cross-sectional study in which participants previously tested in person were recruited to be tested in a remote format. Their sample included 893 children (608 receiving in-person testing and 285 receiving teleassessment), with diagnoses of attention deficit hyperactivity disorder (61%) and anxiety (22%) being most prevalent. Participants were administered select subtests from the WISC-V and/or the Kaufman Test of Educational Achievement-Third Edition (KTEA-3; Kaufman and Kaufman 2014). The results indicated that, for the KTEA-3, there was no difference in performance according to administration mode on the letter and word recognition subtest. On the math concepts and applications subtest, there was a difference in participants tested remotely versus those tested in person, with the latter achieving lower scores, although the effect size was minimal. (Hamner et al. 2022). Results for the WISC-V revealed no difference in scores on the similarities, matrix reasoning, digit span, and vocabulary subtests. A significant difference was observed on the visual puzzles subtest, with those tested remotely scoring higher; once again, however, the effect size was minimal (Hamner et al. 2022). This study contributes to the literature as participants were remotely tested in their natural environment and no proctor was used. In terms of limitations, subtests that required the manipulation of stimuli (e.g., Block Design, Picture Span) were excluded from the study. Thus, it is undetermined how children will perform on subtests that require manipulatives when tests are administered remotely to them in their home environments without a proctor. 

While the literature indicates small differences between remote and in-person scores, these differences should not be taken lightly, particularly in the context of making specific clinical diagnoses or educational classifications using cut scores from psychological instruments. The most illustrative example comes when considering criteria for an educational classification of intellectual disability, where the federal regulations provide general guidance but leave it to individual states to operationalize these criteria. The core criteria are typically an overall IQ score that falls below a certain threshold (e.g., two standard deviations below the mean; McNicholas et al. 2018). A recent study found that most states reference an intellectual deficit; 17 states provide a fixed IQ cutoff, 22 states provide a flexible IQ criterion, and 10 states provide neither (McNicholas et al. 2018). Of note, the authors defined a fixed IQ cutoff as one in which a single IQ score marks the upper bound criterion, above which an individual would not be considered for an intellectual disability (e.g., a score two standard deviations below the mean; McNicholas et al. 2018). In contrast, a flexible cutoff makes reference to a range of scores (e.g., 70–75), to the standard error of measurement or confidence intervals, and to clinical judgment (McNicholas et al. 2018). As many states maintain fixed cutoffs regarding IQ scores for the identification of an intellectual disability, a difference in one point could potentially determine whether a child qualifies for special education services. 

The problem could also manifest in specific learning disability (SLD) identification, more specifically when using the ability achievement discrepancy method (AAD), which is a popular method among school psychologists to identify SLD (Maki and Adams 2019). Under the AAD method, a student is classified with a SLD when they evidence a discrepancy between their cognitive processing ability and academic achievement (Fletcher and Miciak 2019). A full-scale IQ composite is traditionally used as a measure of the student’s overall intellectual ability and various achievement scores are used to determine unexpected underachievement (Kavale et al. 2009). IDEIA does not operationally define the magnitude of the discrepancy in the AAD method, and states have been left to determine their own criteria. The two common methods of identifying a discrepancy are through a regression formula or by calculating the difference between IQ and achievement standard scores (Maki et al. 2015). A total of 34 states currently permit the use of the AAD method, with 13 of them specifying the difference in standard deviation units (i.e., meeting a specific threshold in the difference between IQ and achievement scores) and 11 of them specifying a regression formula (Maki et al. 2015). Fourteen states that allow the use of the AAD method do not indicate a specific discrepancy in identifying SLD (Maki et al. 2015). Regarding the magnitude of the discrepancy, the most common criteria used is a 23-point (1.5 standard deviation units) difference between IQ and achievement standard scores (Reschly and Hosp 2004). Similar to the identification of an intellectual disability, these rigid cut points could mean that one point in either direction could be the difference in a positive SLD classification and the qualification for special education services.

These issues elucidate the importance of the validity and reliability of scores generated by psychological measures. Cognitive and achievement measures are useful (Kudo et al. 2015; Munson et al. 2008; Schneider and Kaufman 2017), but are ineluctably influenced by measurement error; this is as true for comparing the scores between two separate measures as it is between comparing scores of the same measure at different time points (Francis et al. 2005). Aptitude–achievement discrepancy scores can exacerbate errors common to all test scores and render ability–achievement discrepancies unreliable (Barnett and Macmann 1992; Francis et al. 2005; Maki and Adams 2020). These scores have also been demonstrated to be instrument-dependent, as one study found that less than half of the examinees identified with severe underachievement when given the Woodcock–Johnson psycho-educational battery (Woodcock and Johnson 1977) were identified as such when administered the Woodcock reading mastery test (Woodcock 1973; Macmann et al. 1989). Regarding the use of an arbitrary cut score or dichotomizing a continuous variable, classification will be inconsistent because of the measurement error that is pervasive in our instruments (Francis et al. 2005). Even if the differences between remote and in-person assessment scores are trivial, they can still have serious, long-term implications, particularly when making high-stakes educational decisions. Our current instruments, in whatever modality administered, simply do not measure the constructs they purport with the precision necessary to justify rigid cut scores. Practitioners must be aware of score differences across modalities and follow emerging trends in remote assessment moving forward. 

Psychologists are trained to exercise caution when deviating from standardization procedures and test specifications (AERA et al. 2014; Wright and Raiford 2021). However, at what point can the adaptation of assessments via telehealth be considered reliable and valid? Is demonstrating score equivalence enough? These are questions researchers and practitioners are grappling with. A recent survey of school psychologists indicated the provision of telehealth services was one of the most common ethical dilemmas encountered (Maki et al. 2022a). A reading of the literature indicates that there is a lack of consensus regarding the criteria for deeming an adaptation of a test reliable and valid. Wright and Raiford (2021) posit that if equivalence is achieved, scores are interchangeable, and new norms are not needed. Others have advocated more stringent criteria to demonstrate psychometric equivalency, such as equivalency correlations between versions, mean score differences that are not statistically different with small effect sizes, and score dispersion shapes that are not statistically different from one another (AERA et al. 2014; APA 1986; Krach et al. 2020a). Additionally, demographic characteristics from the study sample and the norm sample should be equivalent (Grosch et al. 2011; Hodge et al. 2019; Krach et al. 2020a, 2020b) and the sample size should meet requirements to achieve the statistical power needed to perform equivalency analyses (Cohen 1988; Farmer et al. 2020b; Krach et al. 2020b). Finally, an investigation of the test’s internal structure (typically through exploratory and confirmatory factor analytic techniques) is an essential component of an instrument’s validity as these provide psychometric rationale and justification of the scores produced (Keith and Kranzler 1999; McGill et al. 2020).

It should be noted that the aforementioned equivalency studies discussed above (Hamner et al. 2022; Wright 2018a, 2018b, 2020) do not meet a majority of these criteria. Practitioners should keep in mind if they are interpreting scores for multiple purposes that each purpose must yield validity (e.g., for making a diagnosis or describing a functional level). It is not the test itself, but the interpretive practice that must be validated (AERA et al. 2014). A review of the literature on the reliability and validity of cognitive measures designed for face-to-face administration indicates serious psychometric shortcomings. Nonetheless, practitioners continue to interpret scores in a manner that does not align with the research (Kranzler et al. 2020). Independent investigations of popular cognitive measures have shown problems with longitudinal stability (Styck et al. 2019; Watkins and Canivez 2004; Watkins et al. 2022; Watkins and Smith 2013) and structural validity (Canivez et al. 2017; Dombrowski et al. 2017, 2018; McGill and Spurgin 2017). Additionally, studies examining the diagnostic utility of certain interpretive practices (i.e., Profiles of Strengths and Weaknesses) have consistently produced negative results (Kranzler et al. 2016, 2019; Maki et al. 2022b; Miciak et al. 2014; Stuebing et al. 2002, 2012). 

It is our position that demonstrating score equivalency is insufficient and standardization procedures and norms should undergo a more rigorous process, (see section below on new contributions to the field of remote assessment). Shortcomings in reliability, validity, and diagnostic utility of identifying children with disabilities serve as a cautionary tale within the field of assessment. Prevalent methods of interpretation of cognitive and achievement tests have become so widely accepted and used that it has been challenging to walk them back despite their glaring limitations. As the practice of teleassessment grows, researchers and clinicians should refrain from making assumptions about the capabilities of these technologies. While some organizations (APA 2020) have advised practitioners to use their knowledge or clinical judgment to determine whether scores are an accurate representation of the individual’s functioning, this is challenging enough when tests are used in the manner they are intended to be. Reliance on clinical judgment may open the door to its own fallibilities, as has been well-documented in the clinical assessment literature (Dawes 1996; Dawes et al. 1989; Garb et al. 2016). The advantages of remote assessment are tempting; however, it is important that practitioners allow these technologies to develop, lest we open Pandora’s Box, which has already happened with traditional, in-person assessments. The field should learn from past mistakes and adhere to Weiner’s (1989) maxim: “(a) know what their tests can do and (b) act accordingly” (p. 829). 

## 6. Significant and New Contributions to the Field of Remote Assessment

A promising assessment, and the first normed as a remote assessment, is the Kaufman Brief Intelligence Test—2nd Edition -Revised (KBIT-2 Revised; Kaufman and Kaufman 2022a). This assessment is a cognitive screener often used to estimate an individual’s level of verbal and non-verbal ability, gifted screening, and rapid screening of large populations of learners to determine whether they need a comprehensive evaluation (Kaufman and Kaufman 2022b). The KBIT-2-Revised was normed to allow the examiner to choose between in-person or remote administration. All KBIT-2 Revised data were gathered via remote administration and this group comprises half of the normative sample. The other half of the KBIT-2 Revised was obtained by drawing examinees from the original Kaufman Brief Intelligence Test, Second Edition (KBIT-2; Kaufman and Kaufman 2004) norming sample, all of whom were tested using in-person administration. After drawing these examinees from the KBIT-2 sample, the scores were then equated with the KBIT-2 Revised sample using a differential item functioning method, concurrent calibration, and ability estimates (Kaufman and Kaufman 2022b). 

Three studies were conducted to establish equivalence of in-person and remote administration of the KBIT-2 Revised (Kaufman and Kaufman 2022b). At the preschool level (ages 4–5), 34 demographically matched pairs from the KBIT-2 Revised sample were randomly assigned to either in-person or remote administration. The results indicated equivalence between administration modes; the mean differences between administration modes were trivial (ranging from .15 to 1.7) and effect sizes were minimal (ranging from .01 to .16; Kaufman and Kaufman 2022b). A KBIT-2 2020 sample of 262 children (collected to study relations with the 2004 Kaufman Brief Intelligence Test, Second Edition [KBIT-2; (Kaufman and Kaufman 2004)]) aged 6–16 was compared to the remote KBIT-2 revised sample and yielded similar results with mean differences ranging from .01 to .72 and effect sizes ranging from .00 to .09. Finally, a KBIT-2 2017 sample of 108 children (aged 6–89) was compared to the KBIT-2 revised sample and no differences between administration mode were found, with mean differences ranging from .29 to 1.57 and effect sizes ranging from .07 to .11 (Kaufman and Kaufman 2022b). This is the first instrument to use norms that were collected via remote assessment, with a robust sample, which represents a promising blueprint for future remote assessment development. 

Another assessment in development is the Cognitive Assessment System—2nd Edition: Online Version (CAS-2: Online Version; Naglieri et al. forthcoming). This is a full-battery intellectual assessment. Equivalency studies are now being conducted to create norms for the CAS-2 Online Version. This will be the first norm-referenced cognitive assessment, and if successful in its validation, represents a seminal advancement in remote assessment. 

## 7. Social Justice and Ethical Considerations of Remote Assessment

As we celebrated educational access for many children and adolescents through remote assessment, telehealth services, and online learning during the COVID-19 shutdown, we are compelled to think of the students for whom these services were a barrier. There are more than three million students across the U.S. that lack access to either computers or high-speed and reliable internet; this can also be due to the unaffordability of these services (Kinnard and Dale 2020). This impacts the quality of educational opportunities that were not accessible for many children of lower SES; for example, in Fairfield County, South Carolina, more than half the students did not have access to high-speed internet (Kinnard and Dale 2020). This is a clear example of the vast educational inequities across the United States and precludes compliance with fairness, equity, and justice in Guiding Principle I.3 (NASP 2020b). This highlights the disparities in access to technologies that can deny the basic right to education for marginalized children across the country.

There are certain populations of children who may not be good candidates for remote evaluation. Very young children may not have the attention span to be evaluated in this modality. Similarly, children with attention deficit hyperactivity disorder (ADHD) may also have difficulties attending, sitting still, and not being distracted by objects in their home environment (Shore et al. 2018). Children with oppositional defiant disorder, autism spectrum disorder, or other behavioral problems might shut down the computer if they become frustrated or demands are placed on them that they find disagreeable. Lastly, children who have impairments in hearing or vision should be excluded from remote assessment (Luxton et al. 2010, 2012). While there is no clear literature on who the best candidate is for teleassessment, we contend that children, adolescents, and adults who have adequate attention spans, language skills (receptive and expressive), and competency with technology are the most suitable. 

There are times and situations when teleassessment can provide more equity in evaluations. For example, in rural and remote areas of the country, there may not be a qualified evaluator. In these rural and remote areas, many children show wider gaps in their academic skills than children in urban environments (Goss and Sonnemann 2016). Teleassessment has alleviated barriers to accessing psychological services in these rural and remote areas (Hirko et al. 2020; Marcin et al. 2016). In addition to improved access, teleassessment has reduced transportation costs and time barriers (Burns et al. 2017) as some children live far from the nearest evaluator, making the time and costs involved with the trip(s) prohibitive. There may also be situations where the wait time for a psychological evaluation would be detrimental to a child due to continued academic loss and delay of appropriate placement. Although teleassessment is imperfect, we argue there are situations where the need for a psychological assessment should be the set priority regardless of modality. 

Recently, New York State Education Department (NYSED) has begun to collect information by distributing a digital equity survey, which is a short questionnaire that is meant to determine the technological access and equity among the students in New York (New York State Education Department 2023). However, the survey fails to address whether someone in the home is technologically savvy to access, upload, and utilize all digital content expectations. Although this survey is valuable, there is a need for larger-scale initiatives to provide access to digital literacy as well as to technology.

As school psychologists, we have an ethical obligation to conduct comprehensive evaluations that are equitable and unbiased (Stifel et al. 2020); part of this is to make sure we are using assessments in the way they are intended to be used, which, for most traditional cognitive and achievement tests, is in-person administration. Comparatively, there is a scarcity of teleassessment measures; therefore, during the height of the pandemic the largest district in NY used neither traditional nor remote assessment methods. Instead, school psychologists relied on “comprehensive data-driven assessment” which consisted of data review, interpretation and analysis, teacher reports, and observations. The school psychologist was then tasked with writing a report documenting the eligibility determination of the student suspected of having a disability (R. Deverteuil, C. Joseph and A. Wood, personal communication, 23 February 2023). This assessment method was clearly insufficient, as this manner of record review lacks the use of any norm-referenced assessments that enable the comparison of same-aged peers, which precludes the ability to identify both processing deficits, and academic deficits that are required to classify specific learning disabilities under IDEIA. This is similar to our criticism of taking the “T” out of R.I.O.T. 

There are also concerns about test security; are school psychologists able to keep the integrity of the assessment secure? If an assessment is provided remotely, the content of the test becomes vulnerable. An examinee could potentially save parts of the test’s content or record the session in its entirety. Although we recognize this would most likely be a rare occurrence, there are such situations in which there is motivation to secure tests’ content, e.g., gifted testing. In this context, the exposure to the broader public jeopardizes its validity and clinical utility, with additional legal implications for psychologists related to copyright infringement (Gicas et al. 2021). 

Lastly, university trainers in school psychology must adapt and adhere to the explosion in technological growth. If the knowledge-practice gap is ignored, we are in jeopardy of compromising the integrity of our profession (Miller and Barr 2017). School psychology changes significantly with updated editions of tests and newly created assessments. It is imperative for trainers in school psychology to keep up with the breadth and depth of new information so they can return to the classroom to impart this knowledge. It may be difficult for training programs to add remote assessment procedures into an already packed curriculum and purchase the assessments and their corresponding technologies to adequately train future school psychologists. This may leave many newly trained school psychologists unfamiliar with remote assessment procedures; therefore, it will be incumbent on them to seek out additional professional training.

## 8. Recommendations if Using Remote Assessment

Remote assessment is a relatively new way to assess children and adolescents that burgeoned out of necessity due to the COVID-19 pandemic. Although schools are back to in-person activities, and remote assessment is not a current necessity, we do not see this method of assessment losing too much popularity. With the rapid advances in technology, significant improvements including new remote assessments validated for this purpose are on the horizon, e.g., Cognitive Assessment System 2nd Edition (CAS-2: Online Version; Naglieri et al. forthcoming).

The following recommendations are intended to guide school/clinical/neuropsychologists to provide the best experience and success for themselves as the evaluator as well as the examinee. In addition to the table of guidelines from professional organizations, we provide additional recommendations to consider when conducting remote assessments.

1. Rapport may be more challenging to establish in a remote assessment environment (Bornheimer et al. 2022), and every effort should be made to make the individual feel comfortable. Allowing time to chat, especially for children and adolescents, is a good way to break the ice. Asking questions about their interests, or allowing them to show the examiner a favorite toy may also make the child feel more comfortable.
2. Invite the examinee to a session prior to the start of testing, so that the examiner may prepare them for what they should expect. This can significantly allay the fears or anxiety of the unknown. Provide information on the types of activities they will be engaged in and the time expected for the testing session.
3. Practitioners need to be aware of the developmental or cognitive level of the examinee (Bilder et al. 2020) to limit screen fatigue, thereby compromising the results of the assessment.
4. We encourage examiners to frequently check on the examinee throughout testing, to determine their level of comfort and stamina, as well as technology checks to ensure audio and visual are working optimally (Luxton et al. 2014).
5. Although we do not fully endorse the use of remote assessment at this time, especially to make high-stakes decisions about the classification of children for special education services, we acknowledge there are assessments normed and validated for these purposes. Therefore, we encourage practitioners to stay current in professional development as new remote assessments are introduced to market.

## 9. Conclusions and Future Directions 

The COVID-19 pandemic brought greater attention and focus on the true inequities in public education. Of course, distance learning impacted most children across the 50 states, and around the world. However, the quality and quantity of learning varied, and many children suffered academically. Unfortunately, for many, these academic losses were not recouped and primarily affected the nation’s poorest children. Similarly, mass school closures impacted children awaiting psychoeducational evaluations, and re-evaluations, which left timelines unmet, and delayed many children with suspected disabilities’ offers of special education services. Due to the safety needs of children and school staff, many districts turned to teleassessment to help stay in compliance and maintain legal and ethical standards necessary for psychoeducational evaluations. NASP provided guidance, directing school psychologists to maintain integrity when they are assessing students remotely, maintaining these assessments should be administered the way they were developed and validated; and discouraging the use of teleassessment during the pandemic (NASP 2020a). 

Equivalency studies have shown there are small differences between in-person and teleassessment and provided some justification for the use of remote assessment during extenuating circumstances, i.e., the COVID-19 pandemic. However, these studies are insufficient to justify the use of teleassessment in the long term as these instruments were not intended, normed, and standardized for use in this format. Nonetheless, there are promising new assessments that have been normed, standardized, and validated for remote testing, e.g., KBIT-2 revised (Kaufman and Kaufman 2022a) and additional assessments for remote administration are forthcoming, e.g., CAS-II Online Version (Naglieri et al. forthcoming). While these new technologies and assessments have the potential to solidify the validity of teleassessment, practitioners should exercise caution and consult independent research on these instruments moving forward. 

While remote assessment is a growing and developing practice, newly trained school psychologists will inevitably be exposed. It is critical that they keep in mind the integrity and fairness of the assessment they are using. Further training, either in graduate programs or through extensive professional development, should be offered. Lastly, the social justice and ethical concerns surrounding remote assessment discussed in this paper should be considered. We applaud newly developed assessments intended for remote assessment, and the hope is that they have adequate validity and reliability to accurately capture the constructs they purport to measure so that school psychologists can ethically make decisions about students’ special education status using these new technologies. 

## Figures and Tables

**Table 1:** Guidelines on remote assessment from professional organizations.

Professional Organization	Guidelines
APA ^1^	Ensure test security. Be rigorously mindful of data quality. Think critically about test and subtest substitutions. Widen confidence intervals when making conclusions. Maintain the same ethical standards of care as in traditional psychological assessment services.
NASP ^2^	Preparation and training for both the school psychologist and the adult helping the child at home. Assessments should be administered the way they were developed and validated. Any adaptation should have strong evidence that the results from administering the assessment remotely are similarly reliable to in-person administration, and any adaptations are highlighted in the psychological report. Ensure an appropriate and secure platform is used for remote assessment.
Pearson ^3^	Ensure that remote administration is suitable for the examinee as well as for the referral question. Ensure test security. A virtual meeting should take place prior to testing to address issues related to remote administration. A plan for troubleshooting disruptions/technological issues should be in place prior to the start of the assessment. Ensure technical equipment (i.e., internet connectivity, image/screen size, audio considerations, audiovisual distractions, lighting, teleconferencing software, video, peripheral camera or device, screensharing digital components) allows for a valid assessment. The examiner should follow standardized administration procedures as closely as possible. Record disruptions or atypical events that may have affected the administration process and/or results. Review the current research available on equivalence between different modes prior to using remote administration of a standardized assessment with normative data collected via in-person assessment.
IOPC ^4^	Use available resources to develop competency in remote assessment. Be aware of licensure issues before practicing across state lines Adapt the informed consent process to address issues related to teleassessment. Ensure linguistic and cultural competency regarding issues related to teleassessment. Record disruptions or atypical events that may have affected the administration process and/or results. Document limitations of test adaptations when reporting results Be aware of disparities in access to technology and technological literacy. Be cognizant of cultural factors such as educational attainment, level of acculturation, country of origin, and socioeconomic status when selecting tests. Use HIPPA complaint platforms. Ensure technological equipment allows for a valid assessment.

Note 1. APA = American Psychological Association; NASP = National Association of School Psychologists; IOPC = Inter Organizational Practice Committee. Note 2. APA (2020) ^1^; NASP (2020a) ^2^; Pearson (2021) ^3^; Bilder et al. (2020) ^4^.

## Data Availability

Not applicable.
